# Supplementary material for: PRR14 organizes H3K9me3-modified heterochromatin at the nuclear lamina
Source: Nucleus. 2023 Jan 12;14(1):2165602. doi: 10.1080/19491034.2023.2165602 (PMC9839372; doi:10.1080/19491034.2023.2165602)
Supplement: Supplemental Material [file KNCL_A_2165602_SM3478.docx]

Supplementary Materials for

**PRR14 organizes H3K9me3-modified heterochromatin at the nuclear lamina**

Anna A. Kiseleva, Yu-Chia Cheng, Cheryl L. Smith, Richard A. Katz, Andrey Poleshko*

*Corresponding authors. Email: [poleshko@pennmedicine.upenn.edu](mailto:poleshko@pennmedicine.upenn.edu)

**Supplementary file includes:**

Figs. S1 to S15


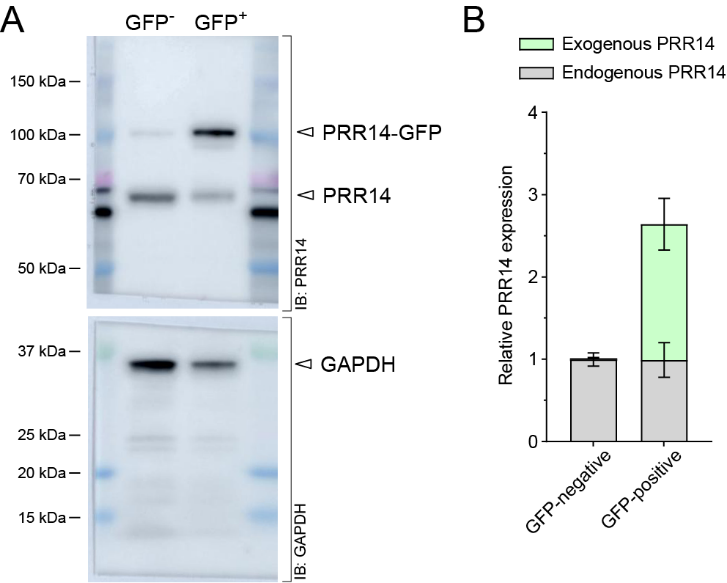


**Figure S1.** **Expression level of exogenous GFP-tagged PRR14 compared to endogenous PRR14 level.** Representative Western blot images of protein lysate from NIH/3T3 cells transfected with PRR14-GFP construct (as in Figure 1) and sorted to obtain separate populations of GFP-negative (GFP-) and GFP-positive (GFP+) cells. Whole cell lysates were then subjected to Western blot with anti-PRR14 and anti-GAPDH antibodies. **(B)** Bar graphs represent quantification of endogenous PRR14 and PRR14-GFP signals from panel A normalized to GAPDH loading control and displayed as expression relative to the level of endogenous PRR14. Error bars show range, n=2.


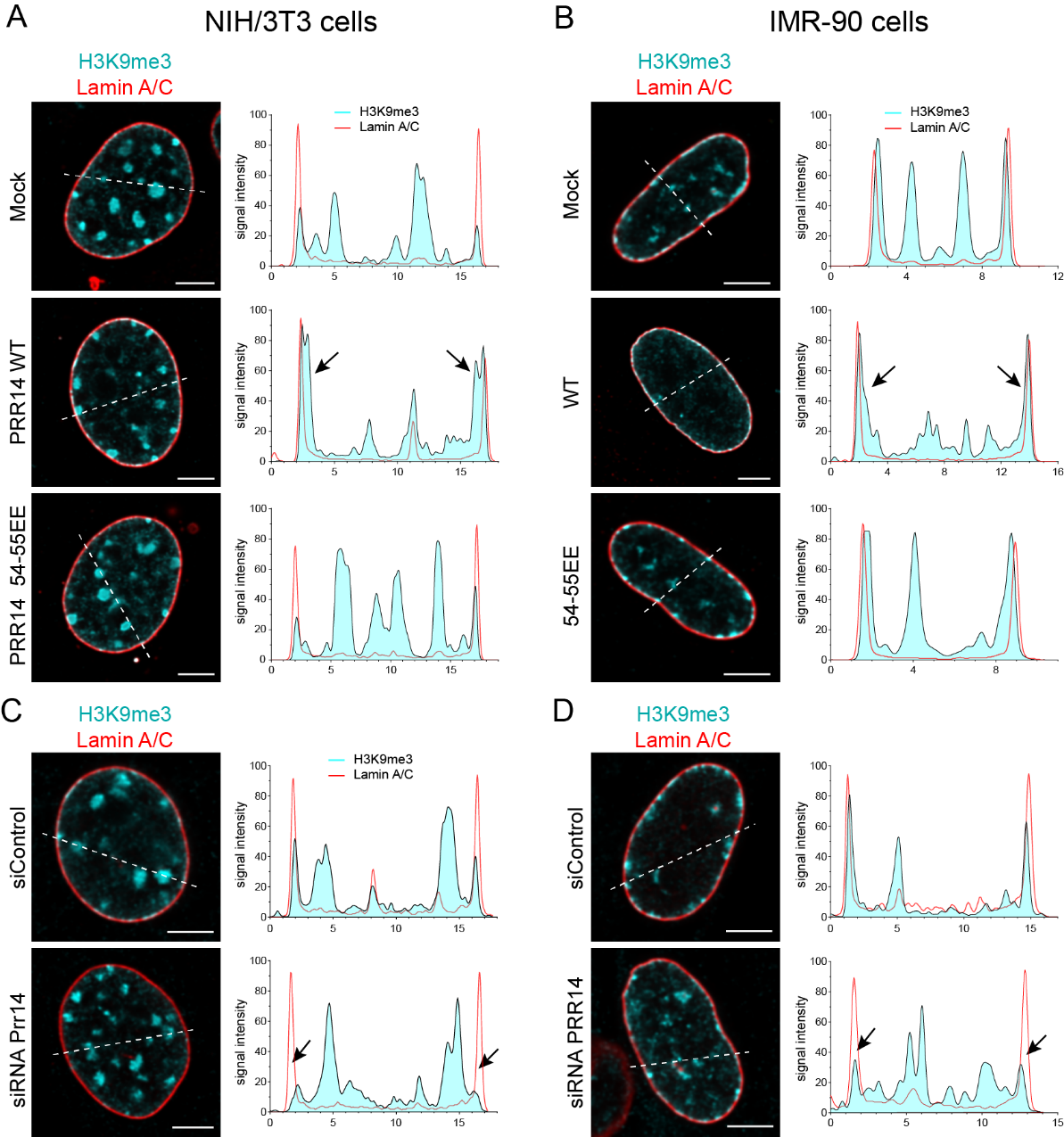


**Figure S2. PRR14 expression influences the amount of H3K9me3-modified heterochromatin at the nuclear lamina.** Representative confocal images, also shown in Figure 1C-D, of **(A)** murine NIH/3T3 and **(B)** human IMR-90 cells expressing only endogenous PRR14 (Mock) or WT or 54-55EE mutant GFP-tagged PRR14 constructs were stained for H3K9me3 (cyan) and Lamin A/C (red). Line graphs show signal intensity line profiles of the H3K9me3 (cyan) and Lamin A/C (red) signal across the dotted lines. Arrowheads show repositioning of H3K9me3-modified heterochromatin to the nuclear lamina. **(C-D)** Representative confocal images of **(C)** murine NIH/3T3 and **(D)** human IMR-90 cells transfected with control or PRR14 siRNAs stained for H3K9me3 (cyan) and Lamin A/C (red), also shown in Figure 1E-F. Line graphs show signal intensity line profiles of the H3K9me3 (cyan) and Lamin A/C (red) signal across the dotted lines. Arrowheads show repositioning of H3K9me3-modified heterochromatin away from the nuclear lamina. Scale bars 5μm.


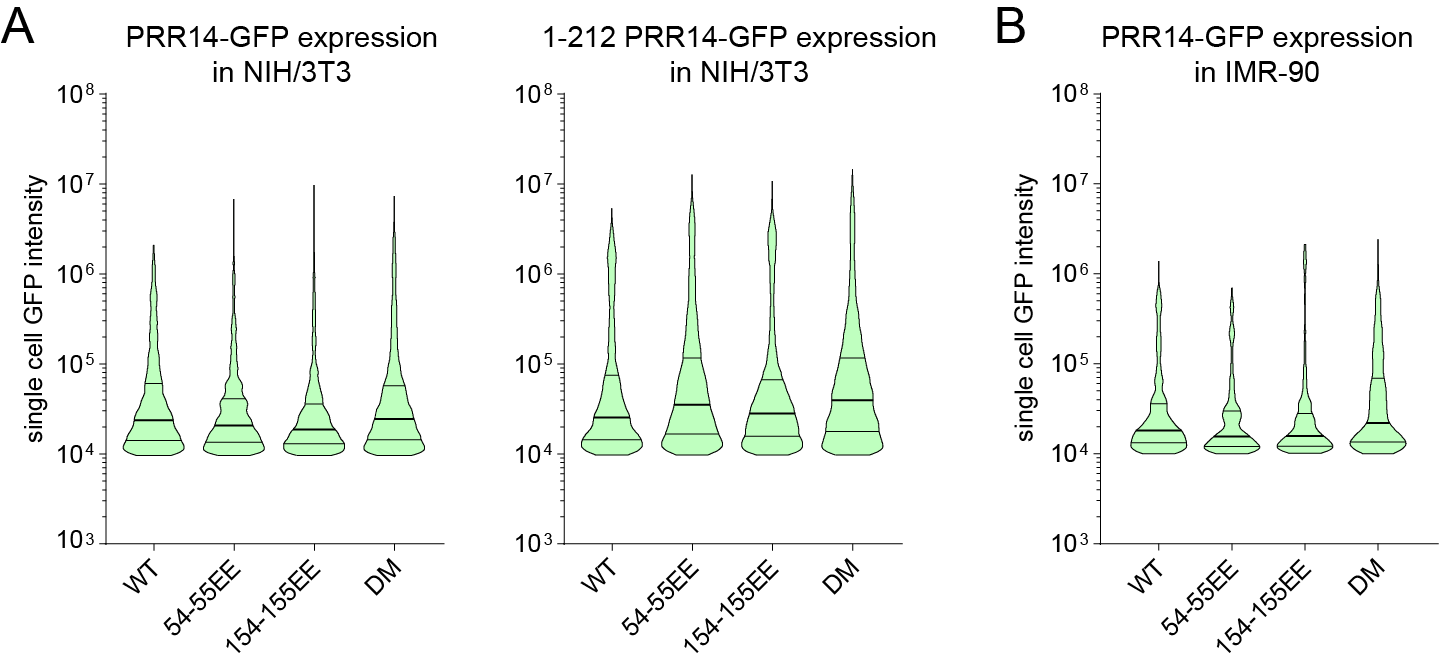


**Figure S3. Expression levels of GFP-tagged PRR14 constructs in NIH/3T3 and IMR-90 cells.** Minimal expression differences were observed between PRR14 constructs which did not correspond with the observed effects on heterochromatin localization, as reported in Figure 1 and Figure S4. NIH/3T3 and IMR-90 cells were transfected with the indicated constructs. Violin plots show distribution of GFP intensities from individual cells expressing indicated GFP-tagged full-length and 1-212 PRR14 constructs in **(A)** NIH/3T3 and **(B)** IMR-90 cells. Lines of violin plots show the median and the interquartile range.


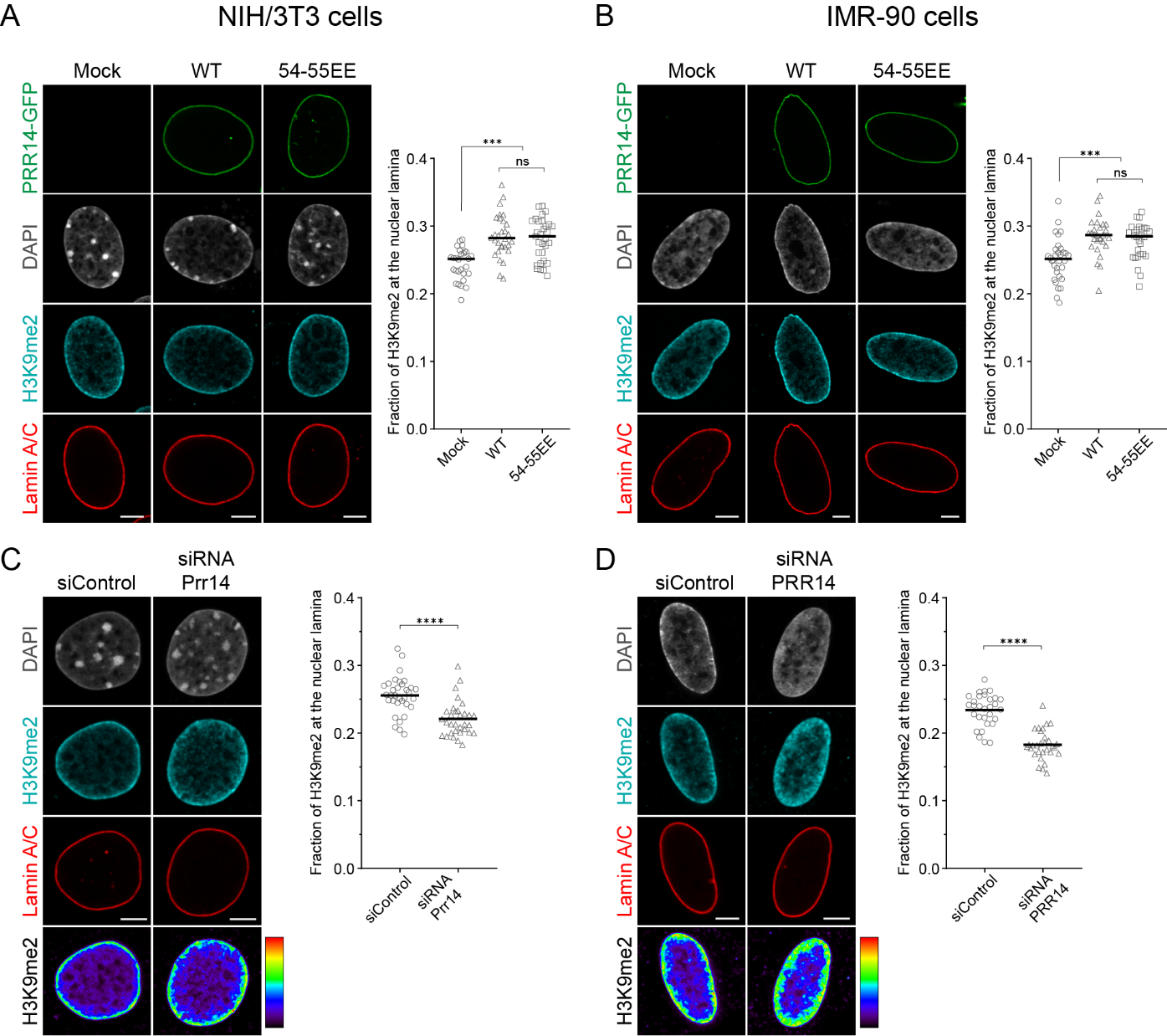


**Figure S4. PRR14 affects H3K9me2-modified heterochromatin at the nuclear lamina.** Representative confocal images of **(A)** murine NIH/3T3 and **(B)** human IMR-90 cells expressing WT or 54-55EE mutant GFP-tagged PRR14 constructs (green), stained for H3K9me2 (cyan) and Lamin A/C (red). **(C-D)** Representative confocal images of (C) NIH/3T3 and (D) IMR-90 cells transfected with control or *Prr14*-specific siRNAs and stained as in panels A and B. DAPI counterstain shown in gray. H3K9me2 signal is also shown as a spectral view in panels C and D for better visualization of the differences. Dot plots show the fraction of H3K9me2 signal at the nuclear lamina. n ≥ 30 cells per condition. Lines on dot plots show median values. Statistical analysis was performed using ANOVA Kruskal-Wallis test with Dunn’s multiple comparisons and Mann-Whitney test; ****p < 0.0001, ***p < 0.001, ns: not significant. Scale bars 5μm.


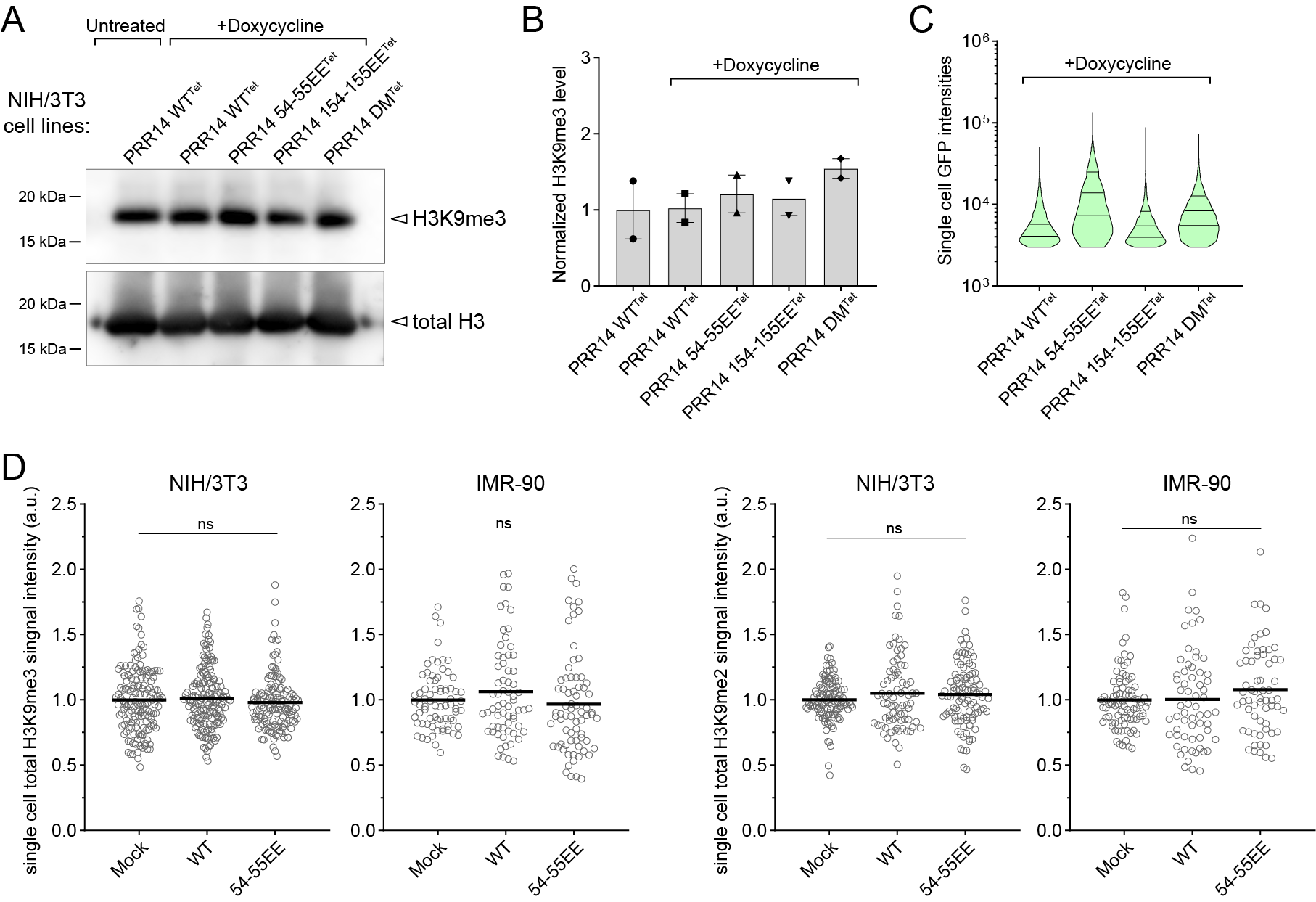


**Figure S5. Amounts of H3K9me3- and H3K9me2-modified heterochromatin do not change upon overexpression of PRR14 constructs**. **(A)** Representative Western blots probed for H3K9me3 and total H3 in protein lysates from untreated and doxycycline-induced NIH/3T3 cells with inducible vectors coding for the indicated PRR14 constructs (PRR14 WT^Tet^, PRR14 54-55EE^Tet^, PRR14 154-155EE^Tet^, PRR14 DM^Tet^). **(B)** Bar graphs show band intensities as a ratio of H3K9me3 to H3 and normalized to the average in the uninduced control cells. Error bars show range. **(C)** Expression levels of indicated GFP-tagged PRR14 constructs in NIH/3T3 cells from panel A. **(D)** Quantification of H3K9me3 (left 2 panels) or H3K9me2 (right panels) immunofluorescent signal from individual NIH/3T3 or IMR-90 nuclei expressing endogenous (Mock) or WT or PRR14 54-55EE. n=62-164 cells per condition. Lines on dot plots show mean values. Statistical analysis was performed using ANOVA Kruskal-Wallis test with Dunn’s multiple comparisons; ns: not significant.


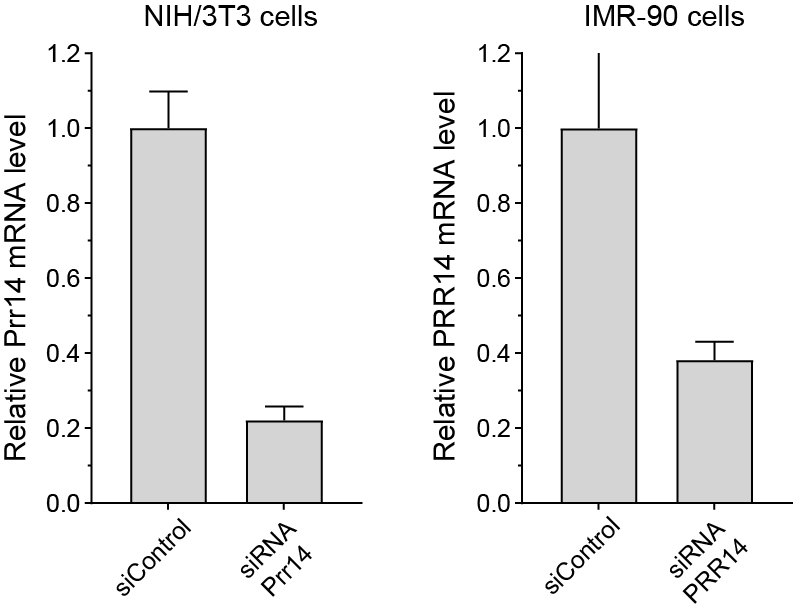


**Figure S6.** Bar graphs show relative levels of PRR14 mRNA in NIH/3T3 (left) and IMR-90 (right) cells 48 hours post transfection with control (siControl) or PRR14-specific siRNA. Error bars show SD, n=3.


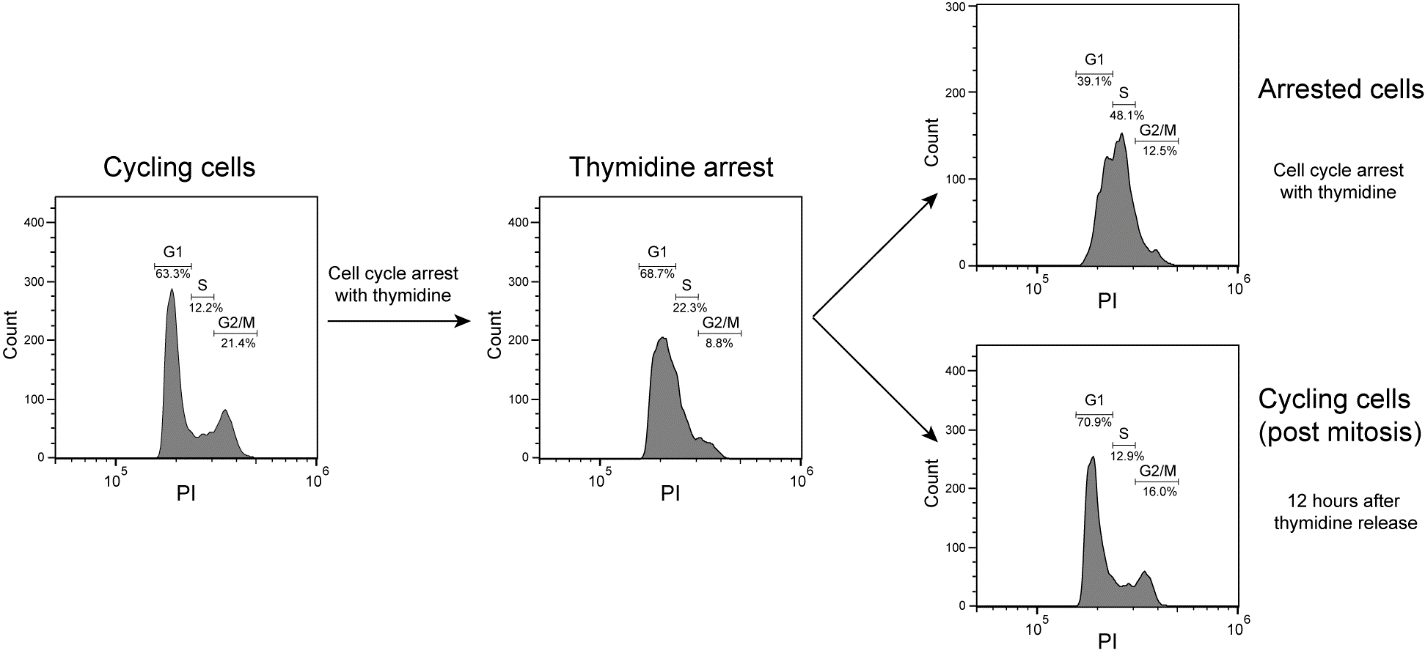


**Figure S7. Flow cytometry charts of cell-cycle arrest with thymidine.** NIH/3T3 cells transfected with PRR14-GFP were arrested with thymidine to prevent mitosis. Samples were maintained in thymidine to prevent mitosis or released from thymidine block 12 hours before fixation to allow mitosis. Flow cytometry charts show normal cycling cells, thymidine-arrested cells, arrested cells 12 hours post-thymidine treatment, and post-mitotic cells 12 hours after thymidine release. G1, S, and G2/M gates show percentage of cells in indicated cell-cycle stages. PI, propidium iodide.


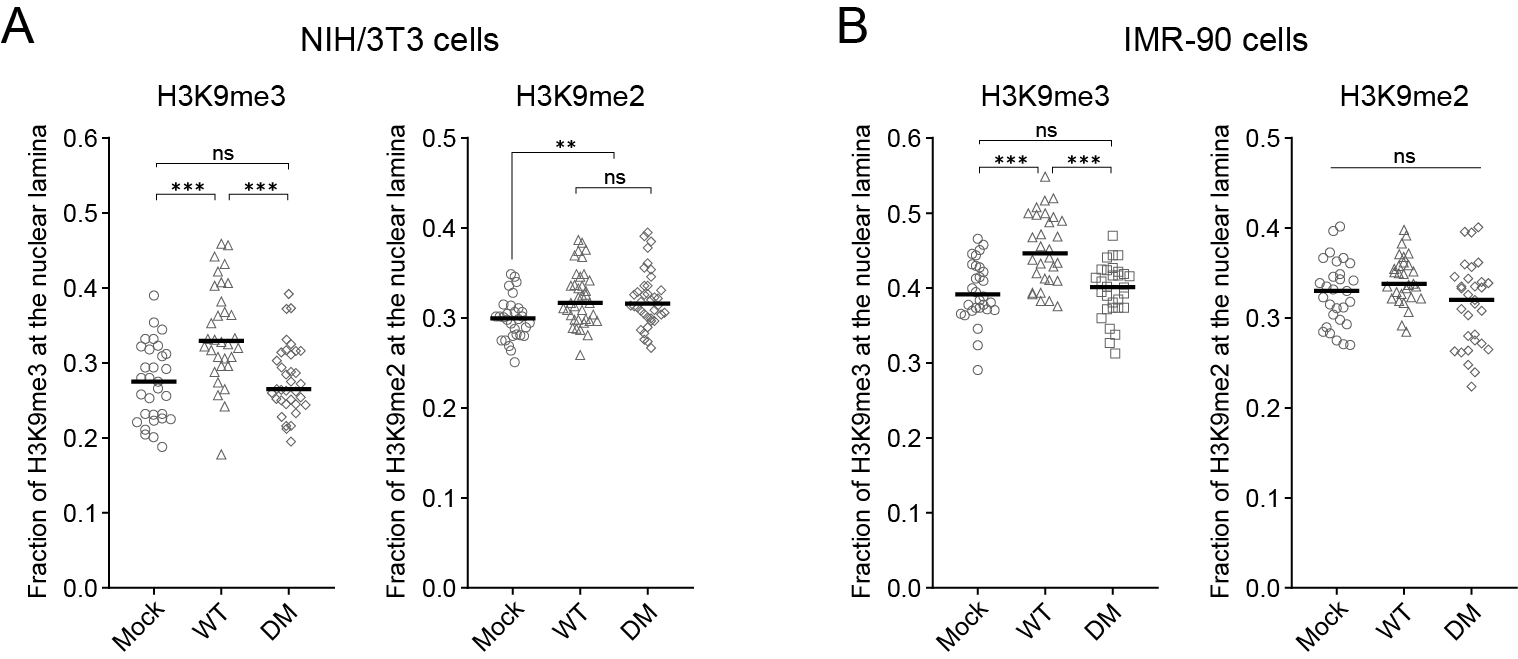


**Figure S8. PRR14 organizes H3K9me3-modified but not H3K9me2-modified chromatin at the nuclear periphery through its HP1 binding sites.** To exclude the possibility that PRR14 HP1-binding site 2 (LVVML) functions to bind and tether H3K9me2 (see Figure S4), we performed the same experiment as in Figure 1 and Figure S4 with PRR14 double mutant (DM) construct (54-55EE and 154-155EE). Dot plots show fractions of H3K9me2- and H3K9me3-marked chromatin at the nuclear lamina in **(A)** NIH/3T3 and (**B**) IMR-90 cells transfected with WT or DM PRR14. n > 35 cells per condition. Statistical analysis was performed using ANOVA Kruskal-Wallis test with Dunn’s multiple comparisons; ***p < 0.001, **p < 0.01, ns: not significant.


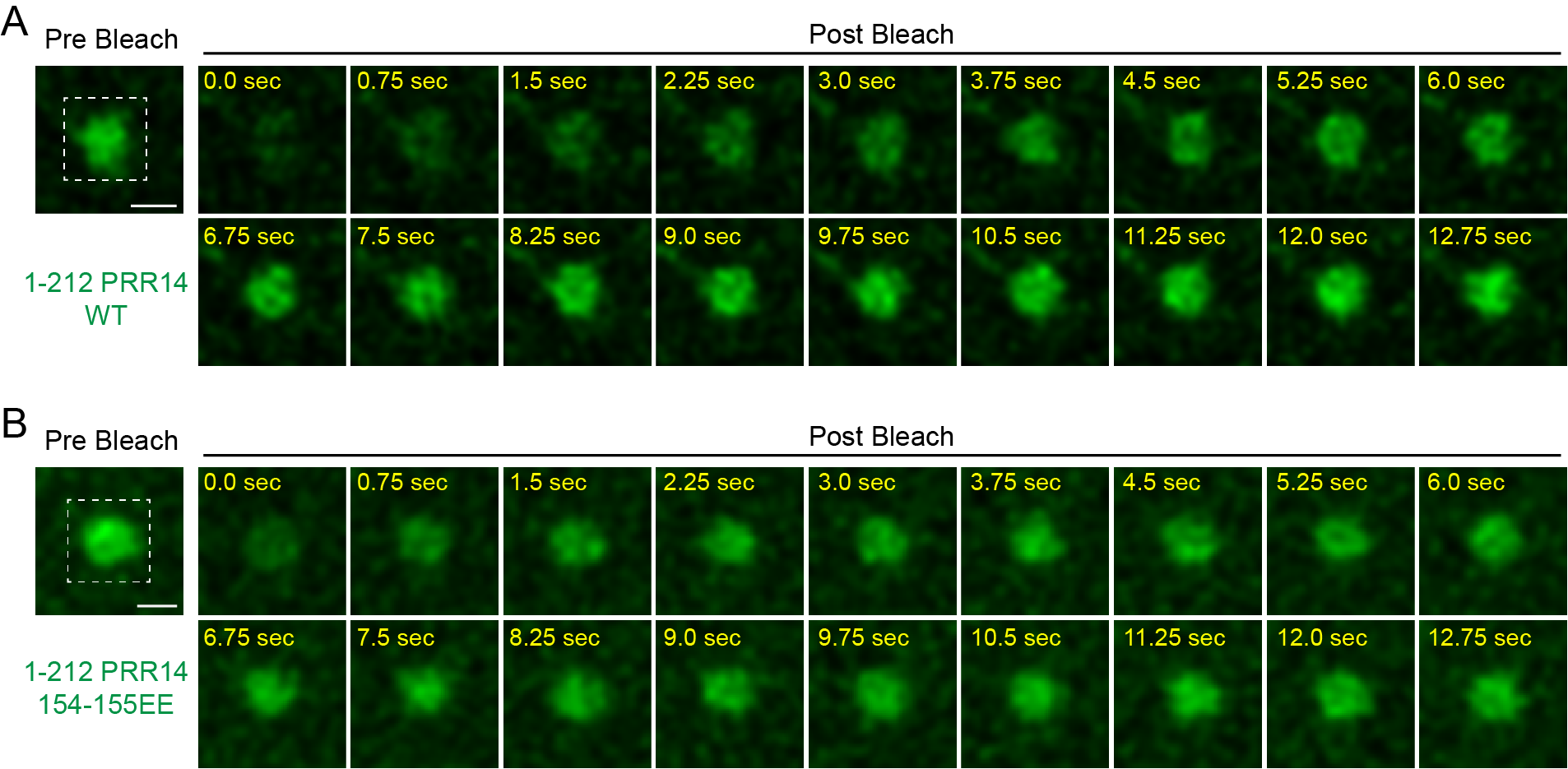


**Figure S9. Representative FRAP images of 1-212 PRR14-GFP constructs.** Zoomed regions of representative confocal images shown in Figure 4 of fluorescence recovery after photobleaching (FRAP) assay of WT 1-212 PRR14 and mutant construct 154-155EE. Scale bars 1μm.


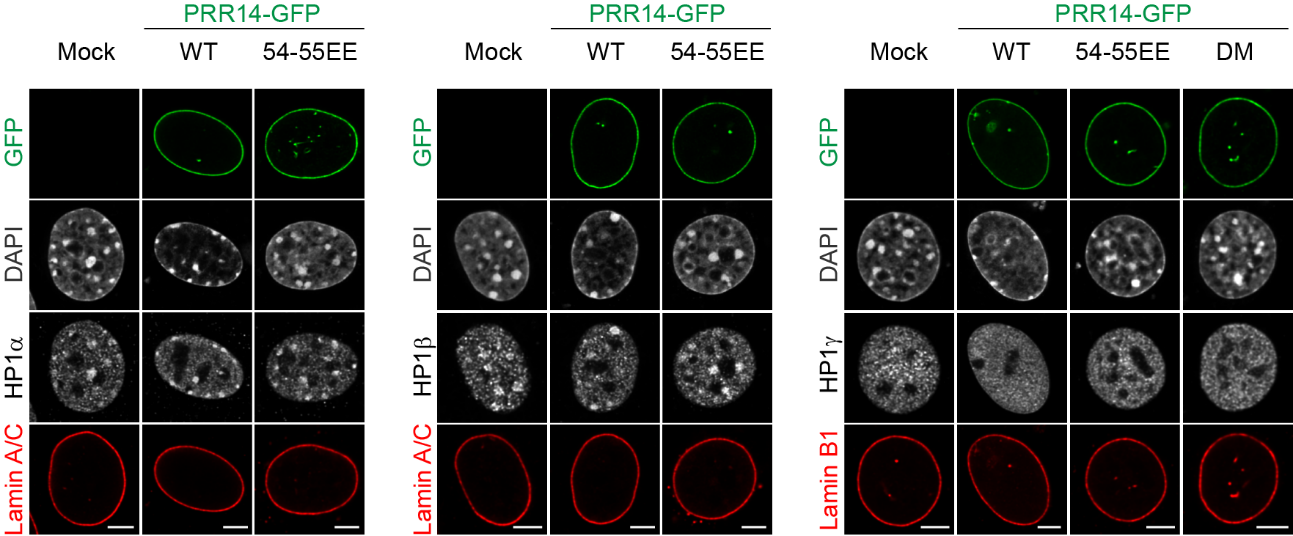


**Figure S10. Overexpression of PRR14 results in repositioning of HP1 proteins towards the nuclear periphery.** Representative confocal images (from Figure 5) show expression of WT PRR14-GFP or mutant constructs and localization of HP1α, HP1β, and HP1γ (in grayscale) in NIH/3T3 nuclei. Scale bars 5μm.


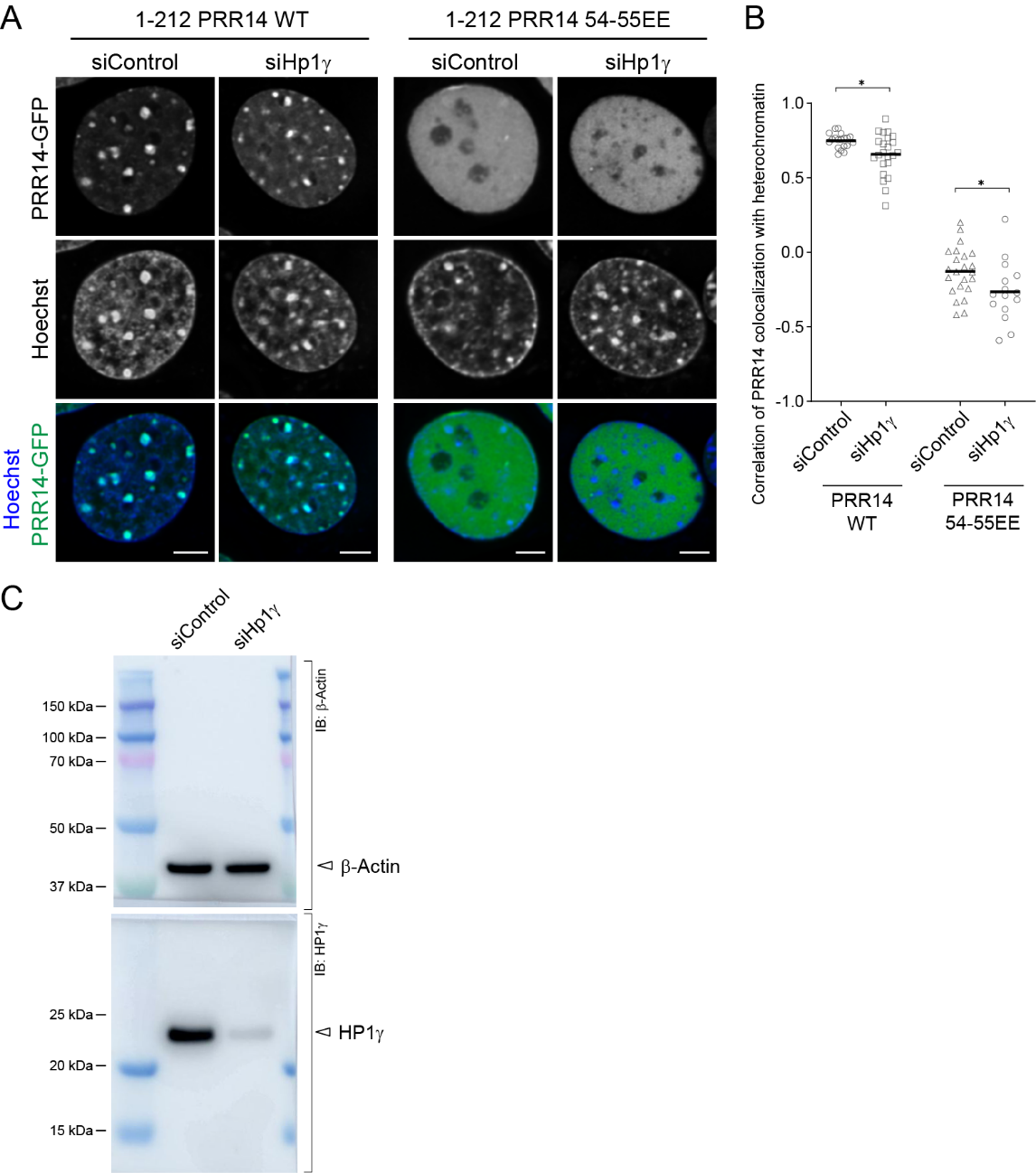


**Figure S11. Knockdown of HP1γ decreases PRR14 efficiency for chromatin binding.** **(A)** Representative confocal images of murine NIH/3T3 cells expressing indicated WT or mutant GFP-tagged 1-212 PRR14 constructs (green), counterstained with Hoechst (blue). Cell with HP1γ knockdown shows increased levels of nucleoplasmic 1-212 PRR14-GFP constructs in cells. Further, 1-212 PRR14 54-55EE construct shows anti-correlation in cells with HP1γ knockdown. **(B)** Dot plots show the Pearson’s correlation of Hoechst staining and GFP-PRR14 signal for each PRR14 construct indicating degree of colocalization of PRR14 with heterochromatin regions. n ≥ 15 cells per condition. Lines on the dot plot show median values. **(C)** Western blots showing HP1γ protein level in NIH/3T3 cells transfected with control (siControl) or HP1γ-specific siRNA (siHP1γ) and β-Actin loading control. IB, immuno-blot. Statistical analysis was performed using Mann-Whitney test; *p < 0.05. Scale bars 5μm.


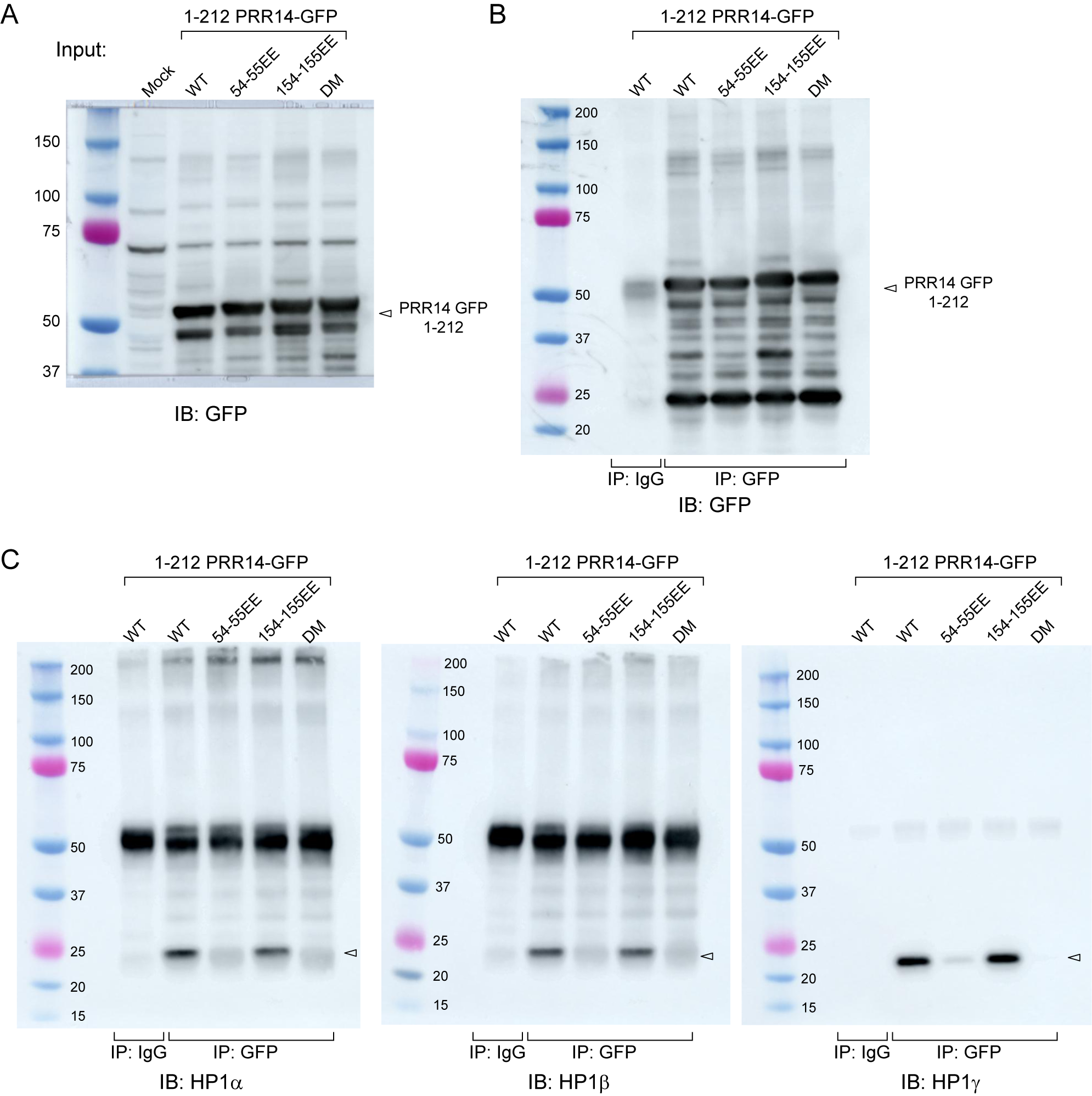


**Figure S12. Full size representative Western blot images for Figure 5.** **(A)** Anti-GFP immuno-blot (IB) of sample inputs for immunoprecipitation experiments from cells that were mock transfected (mock) or overexpressing GFP-PRR14 1-212 fragments (WT or mutants as indicated). **(B)** Immunoprecipitation (IP) of indicated samples with anti-IgG or anti-GFP antibody, immuno-blotted with anti-GFP antibody. Arrowheads in A & B indicate GFP-PRR14 1-212. **(C)** Immunoprecipitation (IP) of indicated samples with anti-IgG or anti-GFP antibody, immuno-blotted (IB) with anti-HP1α (left), anti-HP1β (middle) and anti-HP1γ (right) antibodies. Arrowheads indicate HP1 protein bands.


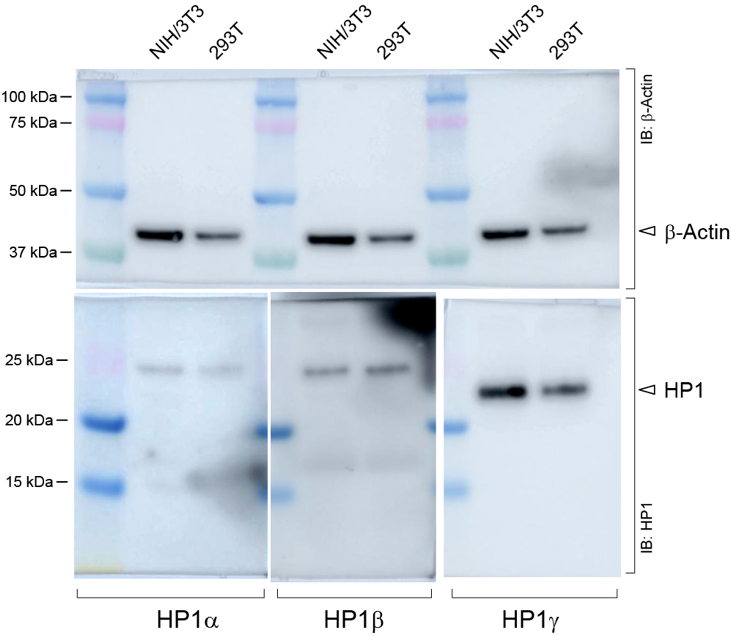


**Figure S13. Expression levels of HP1 isoforms in NIH/3T3 and 293T cells.** Representative Western blot images of protein lysates from NIH/3T3 and 293T cells. Whole cell lysates were then subjected to Western blot with anti-HP1α, anti-HP1β, anti-HP1γ and anti-β-Actin antibodies.


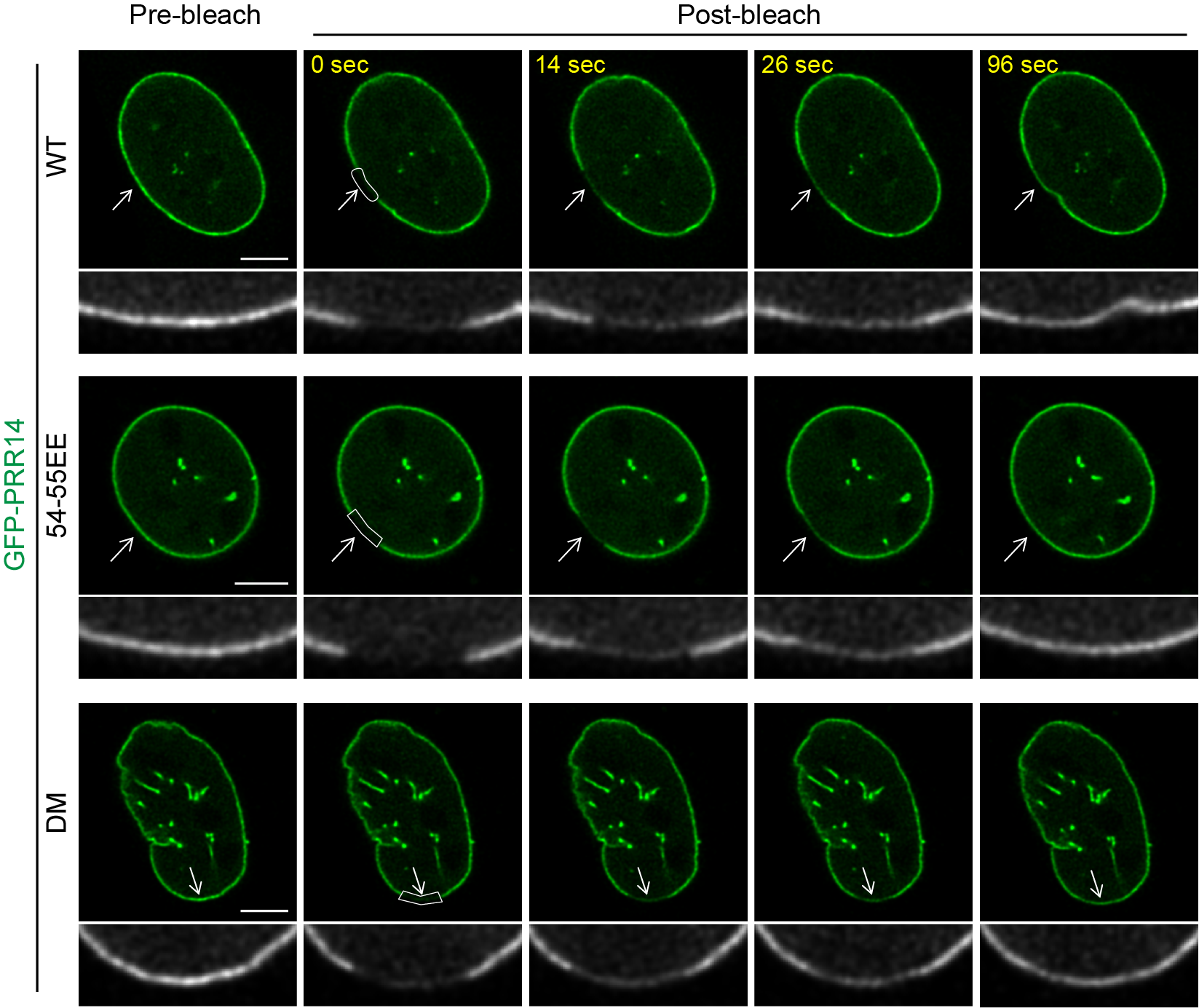


**Figure S14. PRR14 association with the nuclear lamina is independent of PRR14-heterochromatin binding.** Representative confocal images of fluorescence recovery after photobleaching (FRAP) assay of WT full-length PRR14-GFP and mutant constructs in NIH/3T3 cells. DM, double mutant (54-55EE and 154-155EE). Grayscale images show magnified bleached areas, indicated by white arrows. Scale bars 5μm.


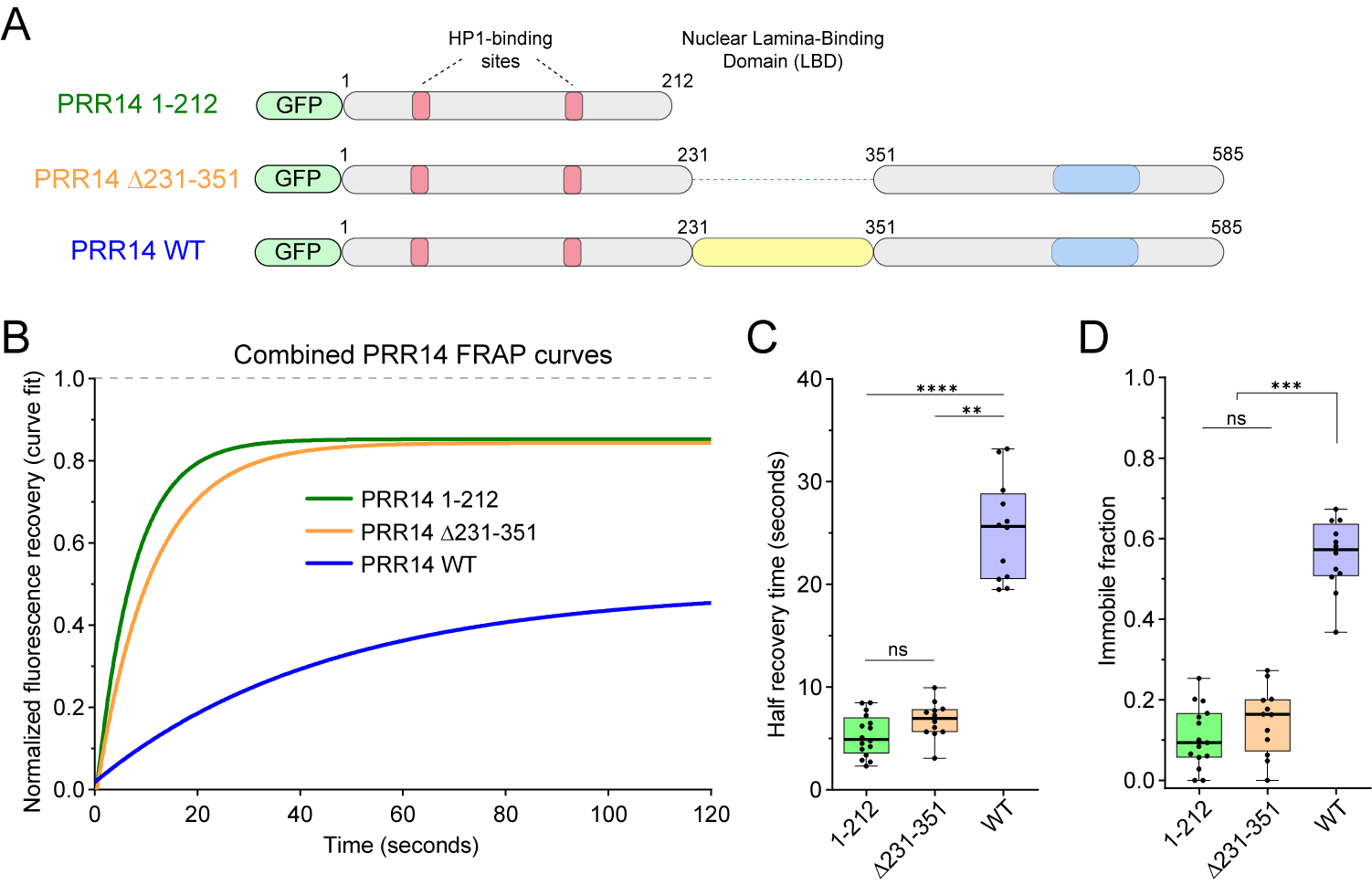


**Figure S15. Non-lamina binding PRR14 shows rapid exchange with heterochromatin, compared to less dynamic full-length WT PRR14 association with the nuclear lamina.** To determine whether the rapid PRR14 dynamics of interaction with heterochromatin result from the smaller size of the GFP-PRR14 1-212 fragment (450 aa) used in the FRAP assay compared to the full-length PRR14 (823 aa), we performed a FRAP assay with a GFP-PRR14 construct that carries a deletion of LBD (PRR14 Δ231-351) which is close in size (703 aa) to the full-length protein. Both PRR14 1-212 fragment and PRR14 Δ213-351 lack interaction with the nuclear lamina and show similar dynamics compared to WT PRR14 protein. **(A)** Schematic representation of PRR14 fragments including N-terminal heterochromatin-binding domain (PRR14 1-212), a nearly full-length version with deletion of the lamina-binding domain (PRR14 Δ231-351), and full-length wild-type protein (PRR14 WT). **(B)** Line graph shows extended curve fits of normalized fluorescent recovery over time after photobleaching of indicated PRR14 constructs. **(C)** Box plots show distributions of recovery half-times for indicated PRR14 constructs. **(D)** Box plots show distributions of immobile fractions for indicated constructs. n ≥ 12 cells per condition. Box plots show median, 25th and 75th percentiles. Whiskers show minimum to maximum range. Statistical analysis was performed using ANOVA Kruskal-Wallis test with Dunn’s multiple comparisons. ****p < 0.0001, ***p < 0.001, **p < 0.01, ns: not significant.
